# Editorial: The problem of childhood hypoglycaemia

**DOI:** 10.3389/fendo.2023.1211933

**Published:** 2023-05-22

**Authors:** Indraneel Banerjee, Klaus Mohnike

**Affiliations:** ^1^ Paediatric Endocrinology, Royal Manchester Children's Hospital, University of Manchester, Manchester, United Kingdom; ^2^ Department of Paediatrics, Otto-von-Guericke-University Magdeburg, Magdeburg, Germany

**Keywords:** hypoglycaemia, children, research, patient, patient – centered care, outcome, congenital hyperinsulinism

Hypoglycaemia is a common problem with significant adverse influence on neurodevelopment in childhood and lasting impact in adult life with consequent neurodisability. Hypoglycaemia is often incompletely understood and inadequately treated. This research topic collates various manuscripts along diverse strands but is unified on the common theme of hypoglycaemia to enrich our current understanding of disease pathways, therapeutic targets and patient impact centred around patient need (**Figure 1**).

**Figure 1:**
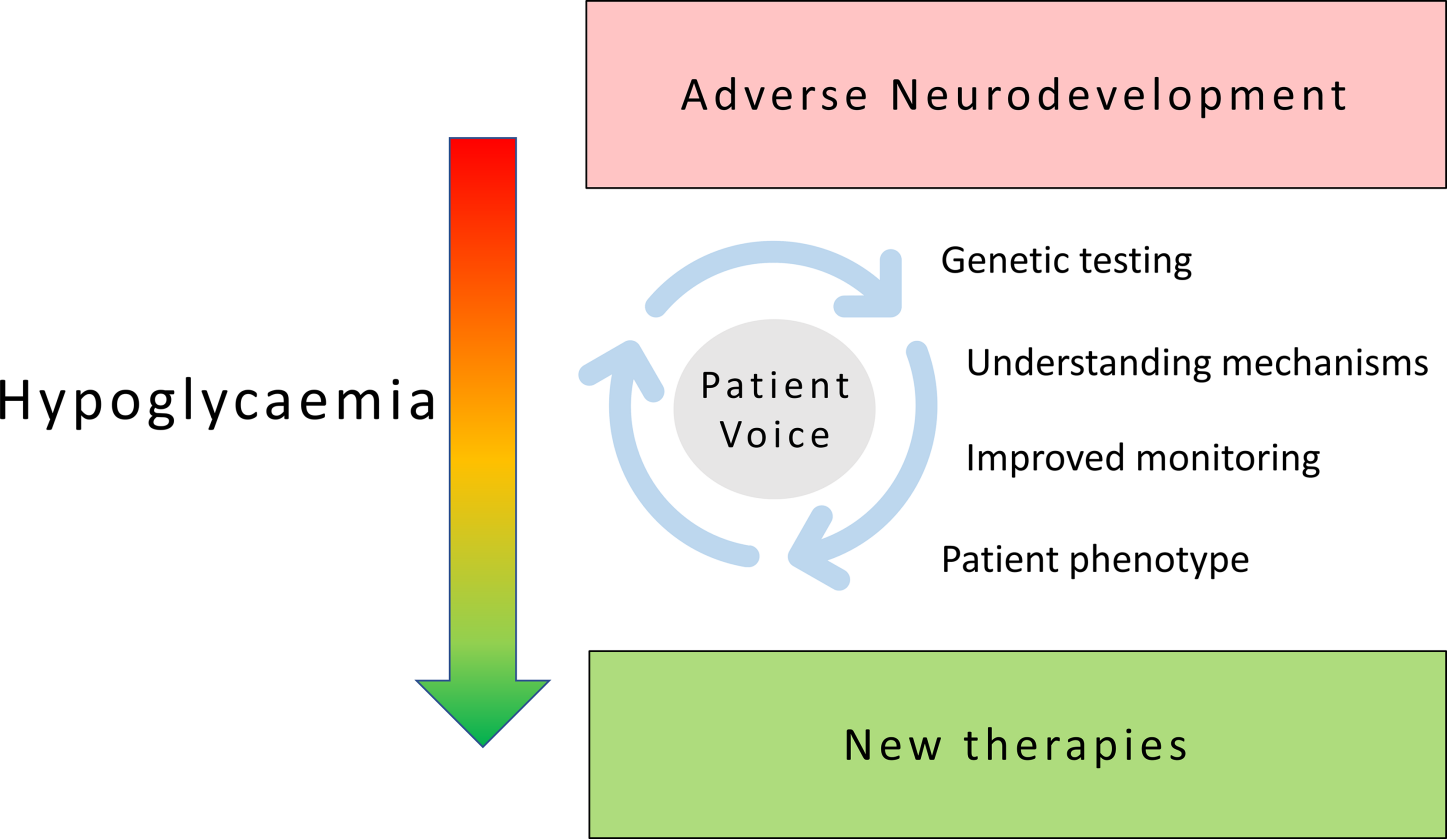
Patient focused research in childhood hypoglycaemia: shifting the burden of neurodisability in childhood hypoglycaemia to new therapies and improved outcomes requires sustained exploration of diverse themes centred around an active patient voice.

Kristensen et al. gets to the heart of the health consequences of hypoglycaemia but concludes that Health related Quality of Life (QoL) studies are infrequent, inconsistent in their reports and often generic to chronic illness, thereby failing to capture disease specific nuances and family perspectives, for example in the case of Congenital Hyperinsulinism (CHI), a disease of severe and recurrent hypoglycaemia in early childhood. The present generation of generic instruments are not sensitive to individual family distress and are therefore unlikely to capture everyday parental anxiety and feelings of despair and helplessness. There is a case to develop CHI specific QoL instruments and apply these to individual aetiologies to draw out meaningful change and derive valid comparisons.

Genetic technology has changed rapidly over the past decade and genetic investigation of hypoglycaemia is no exception. Maiorana et al. discuss gene panel testing in the context of metabolic causes of hypoglycaemia whilst including hyperinsulinism and CHI. Delineating specific genetic association or causation is important to provide certainty of diagnosis without complex and often uncertain biochemical investigations for optimal pharmacologic therapy and for anticipation of prognosis or disease behaviour. Hewat et al. also round up various genetic aetiologies for CHI but suggest caution in simplistic interpretation; phenotypes are variable and a genetic reporting strategy based on previously recognized gene variants may fail to account for the wide variety of seemingly disparate genetic aetiologies and ignore nuances in appreciating pathogenic behaviour. The authors argue for an informed genetic investigational approach utilizing the experience and interpretive abilities of well-established and research active genetic laboratories.

Understanding of genetic aetiology is important not just for the diagnosis of specific forms of hypoglycaemia, such as focal CHI but also for dissecting syndromic forms of CHI described by Zenker at al. The authors explore the strength of association, some being tenuous while others having closer correlation and some having distinctive pancreatic histopathological changes, for example those with Beckwith Wiedemann syndrome. The diversity of genetic aetiology reiterates the heterogeneity of hyperinsulinism in CHI in a nod to Wieland et al. who question traditional understanding of differential gene expressions in the well-recognized form of focal CHI. The authors explore the possibility of integrating pancreatic gene transcript expression to enrich understanding of focal lesional pathobiology. While the recognition of novel genetic correlates may appear to diversify existing heterogeneity, it may promote deeper understanding of pathways for regulation of insulin and potential therapeutic windows in hypoglycaemia.

At present, medical therapies for CHI, a leading cause of childhood hypoglycaemia, are suboptimal; the mainstay of CHI therapy with diazoxide is not applicable to those with mutations in the ATP sensitive K^+^ channel (K-ATP) targeted by diazoxide. van Albada et al. describe alternative therapeutic paradigms in the somatostatin pathway regulating insulin secretion. While somatostatin receptor activation may modulate excess insulin in patients with severe genetic forms of CHI, therapy remains off-label both for short and long-acting forms, although with the promise of newer therapies targeting somatostatin receptor subtypes.

Glucagon like peptide 1 (GLP1) as another molecular target and pathway in CHI amenable to therapeutic modulation, has been reviewed by Danowitz and de Leon. The authors describe the physiology of GLP1 and a similar peptide glucose-dependent insulinotropic polypeptide (GIP), setting the scene for potential therapy with GLP1 receptor antagonists for CHI and post prandial forms of hyperinsulinism. It remains to be seen if such insulin modulating mechanisms have sufficient disease modifying ability to enter the hypoglycaemia treatment repertoire.

A radical departure from traditional investigational methods is described in a paper by Lithovius and Otonkoski. The authors discuss current use of pluripotent stem cells (PSCs) with predicted technical advances leading to the conversion of differentiated PSCs into pancreatic islets to serve as a paradigm changing model exploring novel mechanisms, beta-cell glucotoxicity and novel therapies. While stem cell technologies emanating from research in hyperinsulinism may benefit the understanding of CHI in the first instance, it is likely that collateral information will strengthen our understanding of beta-cell biology and have wider application in other beta-cell disorders such as diabetes.

While molecular technologies advance at pace providing exciting new aetiologies of hypoglycaemia, phenotypic exploration using current investigational tools must also continue, if only to understand the link between abnormal genetic mechanisms and disease manifestations. In the paper by Rossi et al. dynamic methods to characterize hypoglycaemia have been discussed. These range from traditional techniques such as functional *in vivo* testing to more modern *in vivo* metabolic profiling using Continuous Glucose Monitoring (CGM). The paper also discusses the value of isotope tracing to dissect aetiologies in inherited errors of metabolism through the interrogation of metabolites and pathways, for instance in glycogen storage disorder type 1 (GSD1).

There is no substitute to natural history studies in hypoglycaemia, mirroring real-world patient information. Although imperfect with inherent data variability, natural history studies of hypoglycaemia captured in global registries are valuable sources of information to design clinical studies and novel therapies. The topic features a narrative of the development of registries in rare diseases as a whole (Kolker et al.) and more specifically in CHI (Pasquini et al.), a hypoglycaemia disorder with significant family impact. Patient reported data in carefully curated registries are excellent platforms to characterize phenotypic detail that supplement clinician gathered information. As such datasets mushroom, encompassing different groups of patients in different countries, critical evaluation of disease trajectories will no doubt lead to cross correlation of multi-sourced information to build a multidimensional, enriched and individualized model of health need.

A crucial component of the health need in hypopglycaemia is the patient voice, which is often neglected in the face of rapidly expanding technologies and medical opinions. Auckburally et al. take up the patient side to explore the often-unheard voices concurring and dissenting in the use of rapidly emerging technologies such as CGM in hypoglycaemia. Their focused thematic analysis provides a clarion call to adapt and innovate on patient need for optimal health outcomes tailored to the individual. Their manuscript reinforces the increasing importance of the patient perspective in the design of studies and clinical services as joint collaborative enterprises between different disciplines and patient groups, all working harmoniously towards a common goal to alleviate morbidity from debilitating hypoglycaemia.

## Author contributions

All authors listed have made a substantial, direct, and intellectual contribution to the work and approved it for publication.

